# Editorial: The effects of physical activity and exercise on cognitive and affective wellbeing

**DOI:** 10.3389/fnbeh.2022.1047758

**Published:** 2022-11-04

**Authors:** Chong Chen, Suk Yu Yau, Filipe Manuel Clemente, Toru Ishihara

**Affiliations:** ^1^Division of Neuropsychiatry, Department of Neuroscience, Yamaguchi University Graduate School of Medicine, Ube, Japan; ^2^Department of Rehabilitation Sciences, Faculty of Health and Social Sciences, Hong Kong Polytechnic University, Kowloon, Hong Kong SAR, China; ^3^Mental Health Research Centre, Hong Kong Polytechnic University, Kowloon, Hong Kong SAR, China; ^4^Escola Superior Desporto e Lazer, Instituto Politécnico de Viana do Castelo, Viana do Castelo, Portugal; ^5^Instituto de Telecomunicações, Delegação da Covilhã, Covilhã, Portugal; ^6^Graduate School of Human Development and Environment, Kobe University, Kobe, Japan

**Keywords:** physical activity, green exercise, executive functions, working memory, depression, mental health, neuroimaging, neurobiology

A growing body of research suggests that physical activity and exercise enhance a wide range of cognitive and affective wellbeing, including executive functions (Ludyga et al., 2020; Ishihara et al., 2021), memory (Wanner et al., 2020; Aghjayan et al., 2022), creative thinking (Aga et al., 2021; Chen et al., 2021), stress resilience (Arida and Teixeira-Machado, 2021; Belcher et al., 2021), and mental health (Chen et al., 2017; White et al., 2017). Exercise has also been recommended for the treatment of dementia (Cardona et al., 2021) and major depression (Cooney et al., 2013). However, it is still unclear what type, frequency and duration of physical activity and exercise bring the maximal benefits to a specific outcome in a specific population. Furthermore, how findings reported so far can be incorporated into people's everyday life and in educational and psychiatric contexts also remain unaddressed. Finally, the underlying psychological and neurobiological mechanisms of the benefits of physical activity and exercise are still largely unclear. This Research Topic comprises twelve papers that help address these unsolved issues and advance our understanding of the cognitive and affective benefits of physical activity and exercise. Specifically, four important topics emerged from these studies.

Firstly, even a short bout of physical activity or exercise at relatively low intensity may have cognitive and affective benefits. A real-life study by Matsumoto et al. reported that compared to using the elevator, stair-climbing at one's usual pace for three floors roundtrip boosted divergent creative thinking, as assessed by the Alternate Use test. Ando et al. found that both 30 min of aerobic and resistance exercise at a light intensity (40% peak oxygen uptake) reduced participants' reaction time on a Go/No-Go task that measures executive function. However, changes in cognitive performance were not associated with several peripheral biomarkers, including adrenaline, noradrenaline, cortisol, lactate, etc., which calls for further in-depth investigation on other potential mechanisms underlying the cognitive benefits of physical exercise. Physical activity and exercise at low intensities may also improve mental health and have anti-depressant effects. Legrand et al. found that brisk walking for 30 min either in an urban or a green, natural environment reduced participants' negative affect. However, only walking in the green, natural environment increased participants' positive affect, which emphasized the superior benefits of “green exercise” (Chen, 2018; Li et al., 2022). Given that depressed patients often have reduced exercise motivation and physical fitness, Sakai et al. developed an exercise program consisting of 15–25 min of cycling twice a week at an intensity that approaches but never goes higher than subjects' ventilatory threshold (considered light to moderate in intensity). In a pilot study, the authors reported promising therapeutic effects of this program in depressed patients.

Secondly, the effect of high intensity exercise on cognitive performance may depend on the characteristic of exercise and participants. A review by Sudo et al. found that cognitive performance during acute high intensity aerobic exercise is generally impaired while no impairment and even improvement is observed when cognitive tasks are administered over 6 min after high intensity exercise. They also found that cognitive impairment during high intensity exercise is more likely to occur to individuals with low physical fitness and during cycling than running. Age may be another moderating factor but more research is required to reach sound conclusions. The authors also discussed the underlying mechanism of such cognitive-exercise interaction, including regional cerebral blood flow, cerebral oxygenation and metabolism, neurotransmitters, and neurotrophic factors. In contrast to during high intensity exercise, cognitive performance during moderate intensity exercise may be more likely to be enhanced. In a study by Zheng et al., participants stayed sedentary (seating) or exercised on a cycle ergometer at 50% maximal aerobic power for 15 min while simultaneously performed a n-back task and undergone functional near-infrared spectroscopy (fNIRS). It was found that the reaction time for the n-back task was faster in the cycling than seating condition, which was accompanied by reduced concentration of oxygenated hemoglobin in several brain areas, including the dorsolateral prefrontal cortex. Ballester-Ferrer et al. investigated the effects of a 10-week high-intensity functional training program, in which all-out running, jumping rope, or muscle endurance exercise were performed for 10–30 min, 3 times per week. The authors found that while participants in the control group without such training showed no improvement on reaction time on tasks such as the Choice Reaction Test and Interference Test throughout the 10-week period, participants in the training group demonstrated shorter reaction time on these tasks. However, the effect of the training program on psychological wellbeing was absent.

Thirdly, studies have been using mediation analysis to uncover the mechanisms of the benefits of physical activity and fitness. Potoczny et al. found that the effect of Karate training on satisfaction with life was fully mediated by self-control and reappraisal. Hernández-Jaña et al. found that cardiorespiratory fitness and speed-agility fitness but not muscular fitness mediated the association between BMI/central fatness and cognitive performance on eight tasks evaluating working memory, psychomotor speed, and fluid and logical reasoning, etc. Together with evidence that adiponectin, a hormone released by adipocytes, mediates the antidepressant-like and hippocampal neurogenesis enhancing effect of wheel running in mice (Yau et al., 2014), the latter study highlights the interaction between fitness and fatness in influencing cognitive and affective wellbeing.

Fourthly, given that many individuals especially females (Clemente et al., 2016) are physically inactive, there are a number of ways for people to increase physical activity and use physical activity as a strategy to boost cognitive and affective wellbeing in everyday life. As suggested by Legrand et al., one may want to walk to work or walk for one bus stop while commuting and when walk, one may walk to choose greener routes. As suggested by Matsumoto et al., in the workplace, one may want to take the stairs rather than using the elevator whenever possible. Brown and Kwan suggested another strategy, replacing screen time with physical activity. Using isotemporal substitution analysis, the authors found that replacing screen time with moderate-to-vigorous physical activity or sleep is associated with enhanced mental wellbeing. Furthermore, Shen et al. suggests that rather than pure physical activity, activities that simultaneously require cognitive processing may bring greater benefits. The authors found that 8 weeks of Tai Chi Chuan, a mindfulness exercise that tries to integrate the body and mind, improved inhibitory control performance as indicated by reduced reaction time on a flanker task more than that by 8 weeks of brisk walking. Using resting-state functional magnetic resonance imaging (fMRI), the authors found that the improved inhibitory control performance was correlated with spontaneous neural activity in the left medial superior frontal gyrus. Finally, Almarcha et al. suggests that compared to exercise programs prescribed by other people, co-designed exercise programs with inputs from the participants may bring greater benefits. The authors found that whereas a co-designed 9-week exercise program improved self-reported mental health in seven of eight scales used, a prescribed exercise program improved mental health only in three scales.

## Author contributions

All authors contributed to the writing and editing of this Research Topic editorial.

## Funding

CC was supported by a research grant from The Nakatomi Foundation, Japan.

## Conflict of interest

CC is the author of Cleverland: The Science of How Nature Nurtures. The remaining authors declare that the research was conducted in the absence of any commercial or financial relationships that could be construed as a potential conflict of interest.

## Publisher's note

All claims expressed in this article are solely those of the authors and do not necessarily represent those of their affiliated organizations, or those of the publisher, the editors and the reviewers. Any product that may be evaluated in this article, or claim that may be made by its manufacturer, is not guaranteed or endorsed by the publisher.
